# Production and characterization of cell-penetrating recombinant botulinum neurotoxin type A

**DOI:** 10.1016/j.xpro.2022.102026

**Published:** 2023-01-13

**Authors:** Xuan Wei, Jiage Hu, Liling Jiang, Lu Li, Jia Liu

**Affiliations:** 1 Shanghai Institute for Advanced Immunochemical Studies and School of Life Science and Technology, ShanghaiTech University, Shanghai 201210, P. R. China; 2 Shanghai Clinical Research and Trial Center, Shanghai 201210, P. R. China; 3 University of Chinese Academy of Sciences, Beijing, China; 4 Shanghai AsiFlyer Biotechnology Co. Ltd, Shanghai 201210, P. R. China

**Keywords:** Cell culture, Protein Biochemistry, Protein Expression and Purification, Biotechnology and Bioengineering

## Abstract

We recently reported that fusion of cell-penetrating peptides (CPPs) to botulinum neurotoxin type A (BoNTA) proteins could improve the efficiency of cellular uptake. Here, we describe steps to produce and evaluate CPP-BoNTA fusion proteins. We present procedures for the expression and purification of recombinant CPP-BoNTA using insect-cell-based baculovirus expression vector system and *in vitro* characterization of purified proteins. We also detail the analysis of cellular uptake in cell culture and examination of the *in vivo* performance in mice.

For complete details on the use and execution of this protocol, please refer to Wei et al. (2022).^1^

## Highlights

• Production of recombinant cell-penetrating peptide fused-BoNTA (CPP-BoNTA)
• Evaluation of the enzymatic activity of CPP-BoNTA by *in vitro* cleavage
• Evaluation of the cellular uptake of CPP-BoNTA in cell culture
• Evaluation of the *in vivo* performance of CPP-BoNTA in mice

## Abstract (editor highlights)

Publisher’s note: Undertaking any experimental protocol requires adherence to local institutional guidelines for laboratory safety and ethics.

## Graphical abstract

**Figure:**
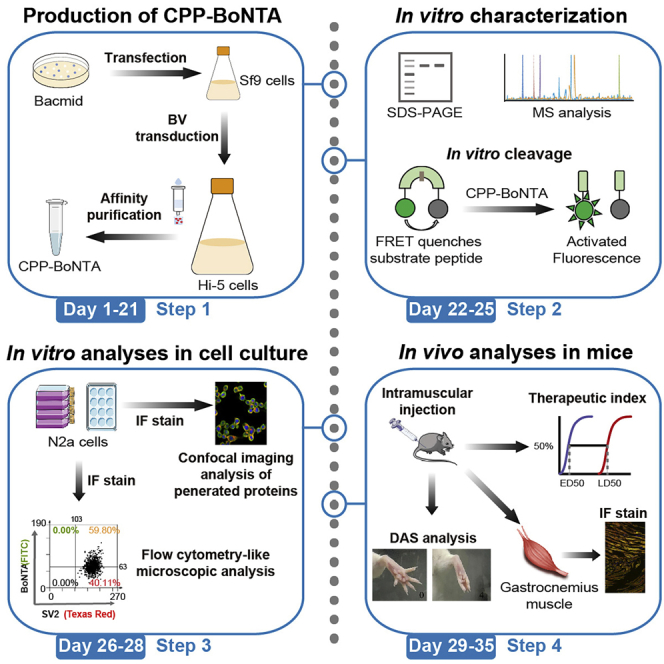

## Before you begin

The following protocol outlines the detailed steps for production and characterization of CPP-BoNTA. A complete list of required materials and equipment is provided in the key resources table. Solutions should be prepared in advance according to the recipes and can be stored as indicated.

We will first describe the procedures and strategies used to optimize the expression and purification of recombinant CPP-BoNTA proteins. Then, we describe the experiments for the characterization of purified proteins including the molecular weight and enzymatic activity. Finally, we describe the procedures for analysis of the activity of proteins on cell culture and in mice.

### Institutional permissions

All mice used in this experiment are housed in specific pathogen-free (SPF) condition with a 12 h light/ 12 h dark cycle (7 AM–7 PM) and have access to food and water *ad libitum* at Shanghai Model Organisms Center (Shanghai, China). All mouse experiments are conducted in accordance with the guidelines of the American Association for the Accreditation of Laboratory Animal Care (AAALAC). All animal experimentation is conducted in accordance with the regulations of Animal Care and Use Committee, Shanghai Model Organisms Center, Inc. For researchers who are about to use this protocol in mice, please acquire permissions from your institutions. All mouse husbandry and experiments should be reviewed and approved by the Laboratory Animal Management and Ethics Committee of your institute and should be in strict accordance with good animal practice as defined by the Laboratory Animal Center of your institute.

### DNA fragments and expression system

Botulinum neurotoxins (BoNTs) are neurotoxic proteins produced by *Clostridium botulinum* and related bacterial species. There are at least seven serotypes and forty subtypes of BoNTs. In addition, several BoNT-like proteins have been reported.^2^ All types of BoNTs block cholinergic neurotransmission by inhibiting the release of acetylcholine.^3^ BoNT type A (BoNTA) is widely used for treating neuromuscular disorders.^4^ Naturally occurring BoNTA is composed of a 100 kDa heavy chain (HC) and a 50 kDa light chain (LC).^5^ The protein sequence of BoNTA in this study can be found from NCBI.

In the past few decades, many expression systems have been developed, such as *Escherichia coli*, yeast, mammalian cells and others. Each system has their own advantages. For example, *Escherichia coli* is known to be affordable and easy to scale up. Mammalian cells can express proteins with post-translational medications. Importantly, insect cells-baculovirus system has proven efficient for expression of complex proteins.^6^ Among the several baculovirus technologies, Bac-to-Bac Baculovirus Expression Vector System (BEVS) provides a rapid and efficient method to generate recombinant baculovirus. The pFastBac1 vector is a suitable vector for both bacterial cloning and protein expression in insect hosts. pFastBac1 contains a polyhedron promoter (P_PH_) to drive robust expression of the gene of interest. A Mini Tn7 element is included to permit site-specific transposition of the transgene into the baculovirus genome.^7^ We synthesize the target gene from a commercial vendor (GENEWIZ) and use molecular cloning technology to insert the target DNA fragment into the pFastBac1 vector.

### Cell lines

Prepare *Spodoptera frugiperda* cells (Sf9) or *Trichoplusia ni* Hi-5 cells (Hi-5) for baculoviral production and protein expression. Prepare mouse neuroblastoma N2a cells or counterparts for evaluation of the cell-penetrating activity of CPP-BoNTA.

### Mice

Prepare 8 to 12-week-old wild-type male or female C57BL/6 mice for intramuscular injection. For each experiment, inject at least five mice per dose.

## Key resources table

**Table:** 

REAGENT or RESOURCE	SOURCE	IDENTIFIER
**Experimental models: Organisms/strains**		

Mouse: C57BL/6J (8–12-week-old male or female)	Shanghai Model Organisms Center	N/A

**Experimental models: Cell lines**		

Neuro-2a (N2a) cells	ATCC	CCL-131

**Antibodies**		

Recombinant Anti-DDDDK tag (Binds to FLAG® tag sequence) antibody (1:200 diluted in 0.2% BSA-PBS)	Novus	NB600-344
Rabbit Polyclonal SV2A Antibody (1:200 diluted in 0.2% BSA-PBS)	Novus	NBP1-82964
Donkey anti-Rabbit IgG (H+L) Highly Cross-Adsorbed Secondary Antibody, Alexa Fluor 568 (1:1000 diluted in 0.2% BSA-PBS)	Life/Invitrogen	A-11055
Donkey anti-Goat IgG (H+L) Cross-Adsorbed Secondary Antibody, Alexa Fluor 488 (1:1000 diluted in 0.2% BSA-PBS)	Novus	NB7539

**Bacterial and virus strains**		

Sf9 cells	Thermo Fisher	12659017
Hi-5 cells	Thermo Fisher	B85502
DH10Bac *Escherichia coli*	Solarbio	C1480
Trelief™ 5α Chemically Competent Cell	TSINGKE	TSC-C01

**Recombinant DNA**		

pFastBac1 vector	Gibco	10360014

**Software and algorithms**		

AKTA pure protein purification system	GE	AKTA pure 25
ImageJ	Schneider et al..^8^	https://imagej.nih.gov/ij/
Peakviewer	SCIEX	https://sciex.com/products/software/peakview-software
Mascot 2.2.1	Matrix Science	http://www.matrixscience.com/
IBM SPSS® Statistics	SPSS Statistics	https://www.ibm.com/cn-zh/products/spss-statistics
ZEN slidescan	ZEISS	https://www.zeiss.com/micro-scopy/en/products/software/zeiss-zenlite.html?vaURL=www.zeiss.com/zen-lite
TissueQuest software	TissueGnostics	https://tissuegnostics.com/pro-ducts/scanning-and-viewing-software/tissuefaxs-imaging-software
GraphPad 6.01	GraphPad	https://www.graphpad.com/dl/96314/10B92408

**Critical commercial assays**		

Gel Extraction Kit	Omega	D2500-02
QIAGEN Plasmid Mini Kit	Qiagen	12123
MinElute PCR Purification Kit	Qiagen	28004
SNAPtide Botulinum Toxin A	Merck/Millipore	567333-200NMOL
Recombinant Botulinum Neurotoxin Type A Light Chain	R&D	4489-ZN-010
Cell Counting Kit-8 (CCK-8)	Dojindo	CK04

**Chemicals, peptides, and recombinant proteins**		

Phanta Max Super-Fidelity DNA Polymerase (1 unit/μL)	Vazyme	P505-d3
*XbaI* (20 units/μL)	New England Biolabs	R0145L
*HindIII* (20 units/μL)	New England Biolabs	R0104L
Cutsmart buffer 10×	New England Biolabs	B7204V
Quick CIP	New England Biolabs	M0525
T4 DNA Ligase	New England Biolabs	M0202L
T4 DNA ligase buffer	New England Biolabs	B0202S
Agarose	TSINGKE	TSJ001
50× TAE Buffer	Sangon Biotech	B548101-0500
GeneGreen Nucleic Acid Stain	TIANGEN	RT210
Trans2K Plus DNA Marker	Transgen	BM111-01
LB Broth Powder	Sangon Biotech	A507002-0250
LB Agar Powder	Sangon Biotech	A507003-0250
Ampicillin	Sangon Biotech	A100339-0025
Kanamycin	Beyotime	ST101
Gentamicin solution	J&K	405947
Chlorotetracycline hydrochloride	J&K	423671
Ethanol absolute	Sinopharm	801769610
Bluo-Gal	Invitrogen	15519028
IPTG	Amresco	0487-100G
Isopropanol	Sinopharm	80109218
FuGENE® HD Transfection Reagent	Promega	E2311
SF-900 II MEDIUM	Gibco	10902088
ESf 921 Insect Cell Culture Medium	Expression Systems	96-001-01
MOPS hemisodium salt	Sigma-Aldrich	M9027
Sodium chloride	Sinopharm	10019318
Glycerol	Sangon Biotech	A100854-0500
Zinc chloride	Sigma-Aldrich	229997
Ni-NTA Agarose	Qiagen	30230
Imidazole	Sangon Biotech	A500529-0100
20× MOPS/ SDS Running Buffer	Sangon Biotech	C506051-0500
ExpressPlus™ PAGE Gel,10 × 8, 4%–20%	GenScript	M42010C
PageRuler ^TM^ Plus Prestained Protein Ladder	Thermo Scientific	26619
eStain L1 Staining Solution, 2.5×	GenScript	M00625R
eStain L1 Destaining Solution, 10×	GenScript	M00626R
HEPES	Gibco	15630080
Botulinum Neurotoxin Type A Light Chain (BoNTA-LC)	R&D	4489-ZN-010
MEM EARLES	Gibco	11090081
MEM NEAA	Gibco	11140050
FETAL BOVINE SERUM NZ ORIGIN	Gibco	10091148
Penicillin-Streptomycin	Gibco	15140122
4% Paraformaldehyde Fix Solution	BBI	E672002-0100
Optimal cutting temperature compound (OCT)	Sakura	4583
Phosphate-Buffered Saline (PBS)	Meilunbio	MA0015
Fetal Bovine Serum (FBS)	Life/Invitrogen	10270106
5% BSA Blocking Buffer	Solarbio	SW3015
HOECHST 33342	Life/Invitrogen	H3570
PROLONG DIAMOND ANTIFADE	Life/Invitrogen	P36970
Carbonate-Bicarbonate Buffer (CBS)	Sigma-Aldrich	C3041
Tween 20	ABCONE	P87875
Poly-L-lysine solution	Sigma-Aldrich	P4707-50ML
Triton X-100	BBI	A600198-0500

**Oligonucleotides**		

BoNTA-F	Genewiz	5′-gcgcgtctagaatggattataaggatgacgacga caagggttccggttcccatcatcaccaccatcacagtt ccggcgtggacctgggtttcgagaacctgtacttcca gggtatgcctttcgttaataaa-3′
BoNTA-R	Genewiz	5′-atataaagcttttacagagggcgttcgcccca-3′
T-BoNTA-F	Genewiz	5′-gcgcgtctagaatggattataaggatgacgacgacaagg gttccggttcccatcatcaccaccatcacagttccggcgtggac ctgggtttcgagaacctgtacttccagggtggtcg taaaaaacgccgc-3′
T-BoNTA-R	Genewiz	5′-atataaagcttttacagagggcgttcgcccca-3′
Z-BoNTA-F	Genewiz	5′-atatatctagaatggattataaggatgacgacgacaaggg ttccggttcccatcatcaccaccatcacagttccggcgtggacc tgggtttcgagaacctgtacttccagggtgaaaaaccgtaca agtgc-3′
Z-BoNTA-R	Genewiz	5′-agtacttctcgacaagcttagccggtatgggtacgttg gtgtgcc-3′

**Other**		

Superdex 200 Increase 10/300 GL	Cytiva	28990944
Assistent Deckglaser Microscopical Circular Cover Glasses	Assistent	92100100030 (01105209)

## Materials and equipment

***Note:*** We suggest using molecular biology grade reagents.

• PCR reaction mix.

**Table:** 

Reagent	Final concentration	Amount
DNA	0.6 ng/μL	1 μL
2 × Phanta Max Buffer	1×	25 μL
Phanta Max Super-Fidelity DNA Polymerase	0.02 units/μL	1 μL
dNTP Mix (10 mM)	0.2 mM	1 μL
Forward primer (10 μM)	0.4 μM	2 μL
Reverse primer (10 μM)	0.4 μM	2 μL
ddH_2_O	N/A	18 μL
**Total**	**N/A**	**50 μL**

• Digestion mix.

**Table:** 

Reagent	Final concentration	Amount
DNA	0.04 μg/μL	1 μL
*Xba*I (20 units/μL)	0.4 units/μL	1 μL
*Hind*III (20 units/μL)	0.4 units/μL	1 μL
Cutsmart buffer 10×	1×	5 μL
ddH_2_O	N/A	Up to 50 μL
**Total**	**N/A**	**50 μL**

• Ligation mix.

**Table:** 

Reagent	Final concentration	Amount
Vector DNA (∼4 kb)	1.2 ng/μL	1 μL
Insert DNA (∼4 kb)	3.6 ng/μL	3 μL
T4 DNA Ligase Buffer 10×	1×	1 μL
T4 DNA Ligase (400 units/μL)	40 units/μL	1 μL
ddH_2_O	N/A	4 μL
**Total**	**N/A**	**10 μL**

• Ni-Lysis buffer.

**Table:** 

Reagent	Final concentration	Amount
MOPS pH7.0 /7.9 (1 M)	20 mM	20 mL
NaCl (5 M)	2 M	400 mL
ZnCl_2_ (10 mM)	10 μM	1 mL
Glycerol	10%	100 mL
ddH_2_O	N/A	479 mL
**Total**	**N/A**	**1 L**

Store at 4°C for 1 week.

• Ni-Wash buffer I.

**Table:** 

Reagent	Final concentration	Amount
MOPS pH7.0 /7.9(1 M)	20 mM	20 mL
NaCl (5 M)	100 mM	20 mL
ZnCl_2_ (10 mM)	10 μM	1 mL
Glycerol	10%	100 mL
ddH_2_O	N/A	859 mL
**Total**	**N/A**	**1 L**

Store at 4°C for 1 week.

• Ni-Wash buffer II.

**Table:** 

Reagent	Final concentration	Amount
MOPS pH7.0 /7.9(1 M)	20 mM	20 mL
NaCl (5 M)	100 mM	20 mL
ZnCl_2_ (10 mM)	10 μM	1 mL
Glycerol	10%	100 mL
Imidazole (5 M)	20 mM	4 mL
ddH_2_O	N/A	855 mL
**Total**	**N/A**	**1 L**

Store at 4°C for 1 week.

• Ni-Wash buffer III.

**Table:** 

Reagent	Final concentration	Amount
MOPS pH7.0 /7.9(1 M)	20 mM	20 mL
NaCl (5 M)	100 mM	20 mL
ZnCl_2_ (10 mM)	10 μM	1 mL
Glycerol	10%	100 mL
Imidazole (5 M)	40 mM	8 mL
ddH_2_O	N/A	851 mL
**Total**	**N/A**	**1 L**

Store at 4°C for 1 week.

• Size exclusion chromatography (SEC) buffer.

**Table:** 

Reagent	Final concentration	Amount
MOPS pH7.0 /7.9(1 M)	20 mM	20 mL
NaCl (5 M)	100 mM	20 mL
Glycerol	10%	100 mL
ddH_2_O	N/A	860 mL
**Total**	**N/A**	**1 L**

***Note:*** The SEC buffer should be freshly made and kept at 4°C until use*.*

• SNAPtide assay buffer.

**Table:** 

Reagent	Final concentration	Amount
HEPES pH 7.4 (1 M)	20 mM	1 mL
Tween 20 (50%)	0.05%	50 μL
ddH_2_O	N/A	48.95 mL
**Total**	**N/A**	**50 mL**

***Note:*** The SNAPtide assay buffer should be freshly made and kept at 4°C until use.

• Cell culture media.

**Table:** 

Reagent	Final concentration	Amount
DMEM	1×	500 mL
FBS	10%	50 mL
Penicillin/streptomycin	100 U mL^−1^	5 mL
Non-essential amino acids	1%	5 mL
**Total**	**N/A**	**560 mL**

## Step-by-step method details

### Production of recombinant CPP-BoNTA proteins

**Timing: 21 days**

This section describes how recombinant CPP-BoNTA proteins are produced. There are three major steps, including plasmid construction (4 days), protein expression (15 days) and protein purification (2 days).

#### Plasmid construction

**Timing: 4 days (for step 1)**

This section describes how to construct recombinant plasmids encoding CPP-BoNTA proteins. Plasmid construction follows the general molecular cloning procedures and includes 6 sequential steps: PCR reaction, restriction digestion, gel extraction of digestion products, ligation, transformation and bacterial cultivation and DNA purification.

1. PCR Reaction.
  a. Set up the PCR reaction using an insect cell codon-optimized synthesized BoNTA gene (GENEWIZ).
    ***Note:*** The forward primers for BoNTA, T-BoNTA and Z-BoNTA (key resources table) all contain N-terminal FLAG and His6 tags (Figure 1A). The PCR primers introduce 5′-XbaI and 3′-HindIII restriction sites for cloning into pFastBac1 vector (Figure 1B).
    **Figure 1:**
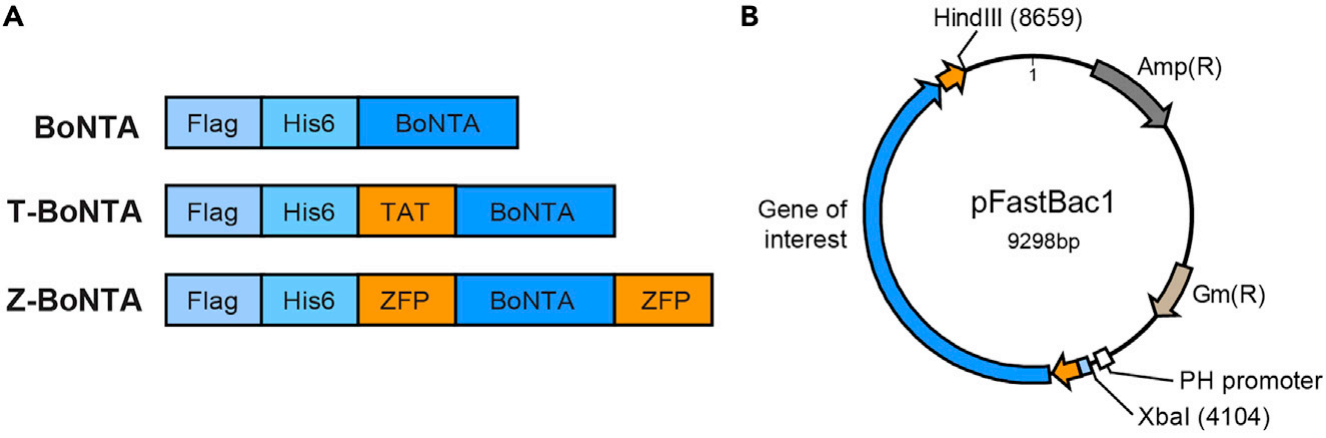
Design of CPP-BoNTA fusion proteins (A) Architecture of BoNTA proteins. The PCR amplicons of BoNTA, T-BoNTA and Z-BoNTA are generated using synthesized BoNTA as the template and different pairs of primers. (B) Plasmid map of BoNTA-harboring pFastBac1 plasmid. The cloning sites for gene insertion is indicated.
  b. Set up PCR reactions in 8-tube strips.
    ***Note:*** Master mix (materials and equipment section) should be prepared for 1.2× the number of samples to account for the loss during pipetting process.
  c. Set up the PCR reaction as follows:

**Table:** 

Steps	Temperature	Time	Cycles
Initial denaturation	95°C	3 min	1
Denaturation	95°C	15 s	30 cycles
Annealing	60°C	15 s	
Extension	72°C	4 min	
Final extension	72°C	5 min	1
Hold	4°C	Forever
  d. Collect the PCR product (Figure 2A) using MinElute PCR Purification Kit (Qiagen). Elute the DNA using 10 μL of water. Store at −20°C for one week.

**Figure 2:**
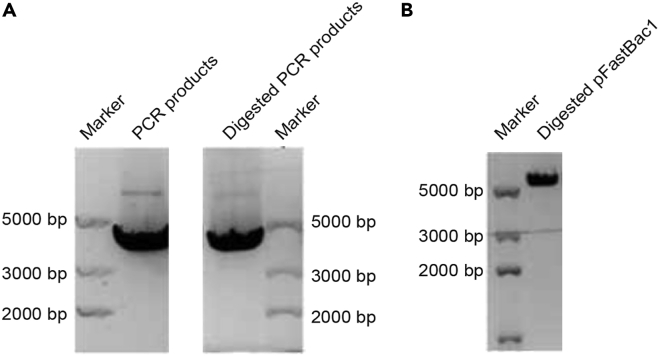
Gel image of PCR products before and after digestion and digested vector (A) PCR product (Marker, Trans2K Plus Marker). The images are collected from separate agarose gels. (B) Digested pFastBac1 vector (Marker, Trans2K Plus Marker).
2. Restriction digestion.
  a. Digest pFastBac1 vector and PCR products with *Xba*I and *Hind*III (Figure 1B).
  b. Set up digestion in 8-tube strips. Prepare master mix according to the table in the materials and equipment section.
  c. Incubate the reaction at 37°C for 3 h, and then add calf intestinal alkaline phosphatase (CIP) of 0.5 μL for further incubation for 30 min.
3. Gel Extraction of digested pFastBac1 vector and PCR amplicon.
  a. Mix the digestion products from step 2 with 10 μL of 6× DNA loading dye.
  b. Prepare 1% (wt/vol) agarose gel with Tris acetate-EDTA (TAE) buffer.
  c. Run the gel at 100 volts (V) for 45 min.
  d. Cut the desired band from the agarose gel, and dissolve it in one volume binding buffer (XP2) for 7 min at 60°C.
  e. Run the solubilized gel through a HiBind DNA Mini Column from the Omega Gel Extraction Kit according to the manufacturer’s instructions, and elute DNA using 20 μL of water.
  f. Store the purified digestion products (Figures 2A and 2B) at −20°C for one week.

4. Ligation.
  a. Ligate the enzyme-treated PCR product and pFastBac1 vector from step 3.
  b. Set up an insert-to-vector molar ratio of 3:1. Master mix can be prepared according to the table in the materials and equipment section.
  c. Incubate the reaction at 16°C for 12–16 h.
5. Transformation.
  a. Thaw 100 μL of Trelief 5α Chemically Competent Cell on ice, and mix them gently with 10 μL of the ligation reaction product from step 4.
  b. Keep the cells on ice for 15 min.
  c. Heat-shock the mixture at 42°C for 90 s using a water bath followed by incubation on ice for 3 min.
  d. Transfer the cell suspension to a culture tube containing 0.5 mL of LB medium.
  e. Incubate the cells with shaking at 200 g for 1 h at 37°C.
  f. Spread 200 μL of the bacterial cell culture on an LB agar plates with 50 μg/mL kanamycin and incubate the plates at 37°C for 12–16 h.
6. Bacterial cultivation and DNA purification.
  a. Prepare growth medium (LB medium containing 50 μg/mL kanamycin). Add 5 mL of growth medium in 14 mL round bottom test tubes.
  b. Inoculate each tube with a single colony from a successful transformation.
  c. Grow bacterial culture for 12–16 h in a 37°C incubator with a shaking platform set to 225 rpm.
  d. Collect the bacteria by centrifugation at 5000 g for 10 min.
  e. Extract the plasmid harboring the gene of interest using the Qiagen plasmid miniprep kit according to the manufacturer’s instructions, and confirm plasmid identity by DNA sequencing.
  f. Store at −20°C for long-term use.

#### Protein expression

**Timing: 15 days (for step 7)**

This section describes the expression of recombinant CPP-BoNTA proteins. The expression process in insect cells relies on BEVS and consists of 5 sequential steps: transposition, bacmid isolation, transfection of bacmid into Sf9 cells, viral amplification and protein expression.

7. Transposition.
  a. Prepare LB agar plates containing 50 μg/mL kanamycin, 7 μg/mL gentamicin, 10 μg/mL tetracycline, 100–300 μg/mL Blue-gal and 40 μg/mL IPTG.
  b. Thaw the DH10BacY competent cells on ice.
  c. Dispense 100 μL of the cells into 1.5 mL tubes.
  d. Add approximately 1 μg recombinant donor plasmid (in 5 μL ddH_2_O) and gently mix the DNA into the cells by tapping the side of the tube.
  e. Incubate the mixture on ice for 30 min.
  f. Heat shock the mixture at 42°C water bath for 45 s.
  g. Chill the mixture on ice for 2 min.
  h. Add 500 μL LB to the mixture.
  i. Place the mixture in a shaking incubator at 37°C with a shaking speed of 225 rpm for 4 h.
  j. Dilute 20 μL transposition mixture with 100 μL LB.
  k. Place 120 μL of each dilution on the plates and spread evenly over the surface.
  l. Incubate for 24–48 h at 37°C.
    ***Note:*** Blue colonies may not be discernible at the first 24 h due to the small size.
  m. Pick white single colony and inoculate into 5 mL LB medium supplemented with 50 μg/mL kanamycin, 7 μg/mL gentamicin and 10 μg/mL tetracycline in 14 mL round bottom test tubes.
  n. Grow at 37°C to stationary phase (16–24 h) with shaking at 250–300 rpm.
8. Bacmid preparation.
  a. For each expression construct, pick up two white colonies for bacmid isolation.
  b. Harvest cells and resuspend cell pellet with 250 μL Buffer P1 from the Qiagen Plasmid Mini Kit and transfer the resuspension into a transparent 1.5 mL Eppendorf tube.
  c. Lyse the cells with 250 μL Buffer P2 followed by neutralization using 350 μL of Buffer N3.
  d. After neutralization, spin at around 16,000 g for 10 min at 4°C.
    **CRITICAL:** Due to the involvement of ethanol during the bacmid extraction, the test tubes should be labeled with ethanol-resistant markers.
  e. After centrifugation, transfer the supernatant (∼800 μL) to a transparent 1.5 mL tube by careful pipetting.
  f. Spin the supernatant at 16,000 g for 3 min at 4°C.
    ***Note:*** This step helps to remove the remaining cell debris, which might decrease the transfection efficiency.
  g. After centrifugation, transfer the supernatant (∼720 μL) to a transparent 1.5 mL tube by careful pipetting. Add 500 μL isopropanol and thoroughly mix the solution.
  h. Spin the mixture for 10 min at 16,000 g at 4°C.
    **CRITICAL:** During this step, set up the transfection experiment in advance by switching on the UV light of the cabinet in the insect cell facility and warming up FuGENE transfection agent (E2311, Promega) to 20°C.
  i. Remove the supernatant by pipetting carefully.
    ***Note:*** A small whitish pellet containing the DNA might be visible at the bottom of the tube.
  j. Add 200 μL cold 70% ethanol to wash the pellet. Spin for 5 min at 16,000 g at 4°C.
  k. After centrifugation, remove the supernatant by pipetting carefully with a 200 μL tip, and then gently add 50 μL cold 70% ethanol.
    **CRITICAL:** The 70% ethanol can keep the DNA pellet and test tubes sterile. Avoid disturbance of the DNA pellet at the bottom of the test tubes when 70% ethanol is added.
  l. Bring the closed tubes containing the bacmids in 70% ethanol into a sterile laminar flow cabinet.
  m. Remove the 50 μL 70% ethanol by pipetting carefully. Air-dry the pellets for 10 min.
    **CRITICAL:** Make sure sterile environment is maintained during this step. Make sure ethanol is completely removed. Residual ethanol in the bacmid samples may affect transfection efficiency and cell viability.
  n. Add 30 μL of sterile water to each tube. Resuspend DNA pellets by tapping gently.
    **CRITICAL:** Due to the large size, improper resuspension may cause damage to bacmids due to shearing force.

9. Transfection of bacmid into Sf9 cells.
  a. Seed 0.5–1.0 × 10^6^ insect cells into a 6-well plate and incubate at 27°C for 15 min before transfection.
    ***Note:*** The 15 min incubation allows the cells to settle.
  b. Prepare the following solution in the sterile cabinet for bacmid transfection:
    i. Solution A: add 10 μL FuGENE transfection agent into 100 μL Sf-900 II serum-free medium.
    ii. Solution B: add 25 μL bacmid into 200 μL Sf-900 II serum-free medium.
  c. Mix solution A and solution B and incubate at 25°C for 15–30 min.
  d. Added mixed solution A-B into seeded Sf9 culture drop by drop.
    ***Note:*** The two bacmid preparations from two separate single colonies as described in step 8 are used in transfection to ensure the success of BV production. Each bacmid has two transfection replicates and a non-transfected group is also included. This corresponds to five transfection group on the plate, including bacmid1, bacmid1’ (replicate), bacmid2, bacmid2’ (replicate) and a control group.
    **CRITICAL:** The final medium volume after transfection should be 2.5–3.0 mL.
  e. After transfection, swirl the 6-well plate gently to ensure even distribution of the cells.
  f. Incubate the transfected 6-well plates at 27°C in the dark. Check the cells every day for contamination and viability.
    ***Note:*** Successfully transfected cells typically exhibit enlarged size in morphology. The majority of the cells will be infected at the third day post transfection.
  g. Collect the medium supernatant at 72 h after incubation.
  h. Centrifuge the medium supernatant at 1,000 g for 10 min at 4°C to remove cell debris.
  i. Harvest the supernatant as initial virus (P1). Store at 4°C in dark for immediate use or at −20°C in dark for long-term use.
    ***Note:*** Baculovirus is regarded as biosafety level 1 (BSL-1) reagent by authority. However, due to the replicative nature of baculovirus in insect cells, it is preferred to handle baculovirus in virus-specialized cabinet to prevent potential cross contamination.

10. Viral amplification.
  a. Seed insect cells with viability of more than 95% into 125 mL shake flasks containing Sf-900 II serum-free medium at a density of 1–1.5 × 10^6^/mL.
  b. Add 2 mL P1 viral stock to suspension Sf9 cells.
  c. Incubate the cells in a 27°C incubator with a stirring rate of 120 rpm.
  d. At 72 h post-infection, count and record cell density and viability.
  e. Collect the medium containing virus (P2) from shaker flasks.
  f. Centrifuge the virus-containing medium at 500 g for 5 min at 4°C to remove cells and large debris. Store at 4°C in dark for immediate use or at −20°C in dark for long-term use.
  g. Seed insect cells with viability of more than 95% into 250 mL shake flasks containing Sf-900 II serum-free medium at a density of 1–1.5 × 10^6^ cells/mL.
  h. Add P2 viral stock (1% v/v) to suspension Sf9 cells.
  i. Incubate the cells in a 27°C incubator with a stirring rate of 120 rpm.
  j. At 72 h post-infection, count and record cell density and viability.
  k. Collect the medium containing virus (P3) from shaker flasks.
  l. Centrifuge the virus-containing medium at 500 g for 5 min at 4°C to remove cells and large debris. Store the supernatant at 4°C in dark for immediate use or at −20°C in dark for long-term use.
11. Protein expression.
  a. Seed Hi-5 insect cells with viability of more than 95% into 1 L shake flask containing 300 mL ESf 921 Insect Cell Culture Medium at a density of 2 × 10^6^ cells/mL.
  b. Add P3 viral stock (1% v/v) to suspension Hi-5 cells.
  c. Incubate the cells in a 27°C incubator with a stirring rate of 120 rpm.
  d. At 48 h post infection, count and record cell density and viability.
  e. Collect the cells by centrifuging the culture at 500 g for 10 min at 4°C to remove supernatant.
  f. Store the cell pellet at −80°C for one year.

**Pause point:** The pellet can be stored at −80°C for one year but prolonged storage is not preferred for purification.

#### Protein purification

**Timing: 2 days (for step 12)**

This section describes the purification procedures of recombinant CPP-BoNTA proteins. Ni-NTA affinity chromatography and size exclusion chromatography (SEC) are involved. Either manual or automatic purification can be performed.

12. Purification with Ni-NTA agarose beads.
  a. Resuspend cell pellet in 50–100 mL Ni-Lysis buffer, followed by homogenization by sonication with the settings of 70% power and two cycles of 5 min treatment with 5 s on/5 s off interval.
  b. Centrifuge the sonicated cells at 16,000 g for 30 min at 4°C.
    ***Note:*** If the centrifuged solution is not clear, transfer the supernatant to a new tube and repeat the centrifugation step.
  c. Transfer the supernatant to a new tube. Add 2 mL Ni-NTA agarose beads (50% slurry; 1 mL settled volume) and rotate at 4°C for 1–3 h.
    ***Note:*** Before mixing the Ni-NTA agarose beads with cell lysates, equilibrate the beads with 5 column volume (CV) of Ni-Lysis buffer.
  d. Transfer the Ni-NTA agarose beads into column and wash with 50 mL Ni-Wash buffer I.
  e. Wash the beads with 50 mL Ni-Wash buffer II. Concentrate the flow through to about 3 mL using 50 MWCO spin concentrator at 4°C.
  f. Wash the beads with 50 mL Ni-Wash buffer III. Concentrate the flow through to about 3 mL using 50 MWCO spin concentrator at 4°C.
  g. Elution with 50 mL Ni-elution buffer. Concentrate the elute to about 3 mL using 50 MWCO spin concentrator at 4°C.
13. Purification with SEC columns.
  a. Equilibrate Superdex 200 resins in 10/300 GL column with 1.5 CV SEC buffer.
  b. Load 500 μL eluted sample onto Superdex 200 in 10/300 GL column.
  c. Harvest the target proteins according to the UV reads.
  d. Store purified proteins at −80°C for one year.

**Pause point:** The protein samples can be stored at −80°C for one year but the activity should be carefully checked after prolonged storage.

### *In vitro* characterization of CPP-BoNTA

**Timing: 4 days**

Before conducting analysis of the activity of CPP-BoNTA, the integrity and purity of the proteins should be evaluated. This section includes the procedures for three major steps: SDS-PAGE analyses, mass spectrometry analyses and *in vitro* cleavage assay.

#### Analysis of purity by SDS-PAGE

**Timing: 1 day**

14. SDS-PAGE analysis of purified CPP-BoNTA proteins.
  a. Pull off the sticker from the GenScript pre-made gel and place the gel in the gel tank.
  b. Transfer 1× MOPS SDS running buffer to the gel tank.
  c. Remove the comb and load 10 μL of protein sample in each well.
  d. Run the gel at 200 V for roughly 30 min.
  e. Stain the gel with Coomassie blue staining buffer for 20 min.
  f. Destain the gel with destaining buffer for roughly 40 min.

**Figure 3:**
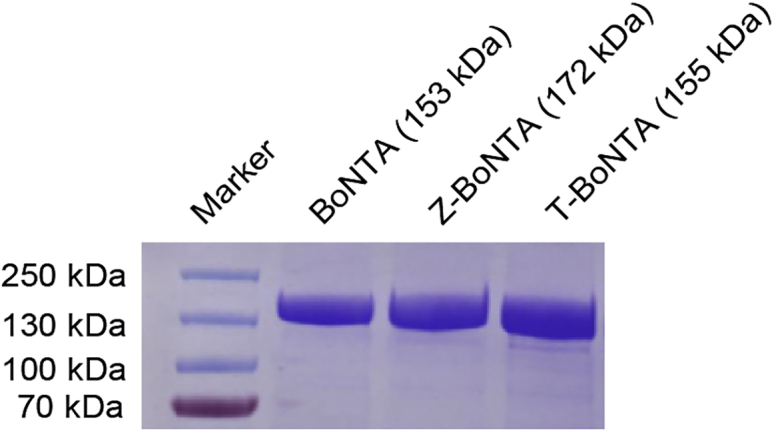
Purified recombinant CPP-BoNTA fusion proteins The molecular weight of each protein is indicated.

A representative result is shown in Figure 3.

***Note:*** This step is mainly used for analysis of the purity of the CPP-BoNTA proteins.

#### Analyses of molecular weight by mass spectrometry

**Timing: 2 days**

***Note:*** Molecular weight is the primary parameter in the identification of peptides and proteins and can be determined by time of flight (TOF) mass spectrometry (MS). The principle of TOF is that ions will accelerate through the flight pipeline under the action of electric field, which will be then detected by the detector according to different flight time. The mass/charge ratio of ions (M/Z) is proportional to the flight time of ions.

**Figure 4:**
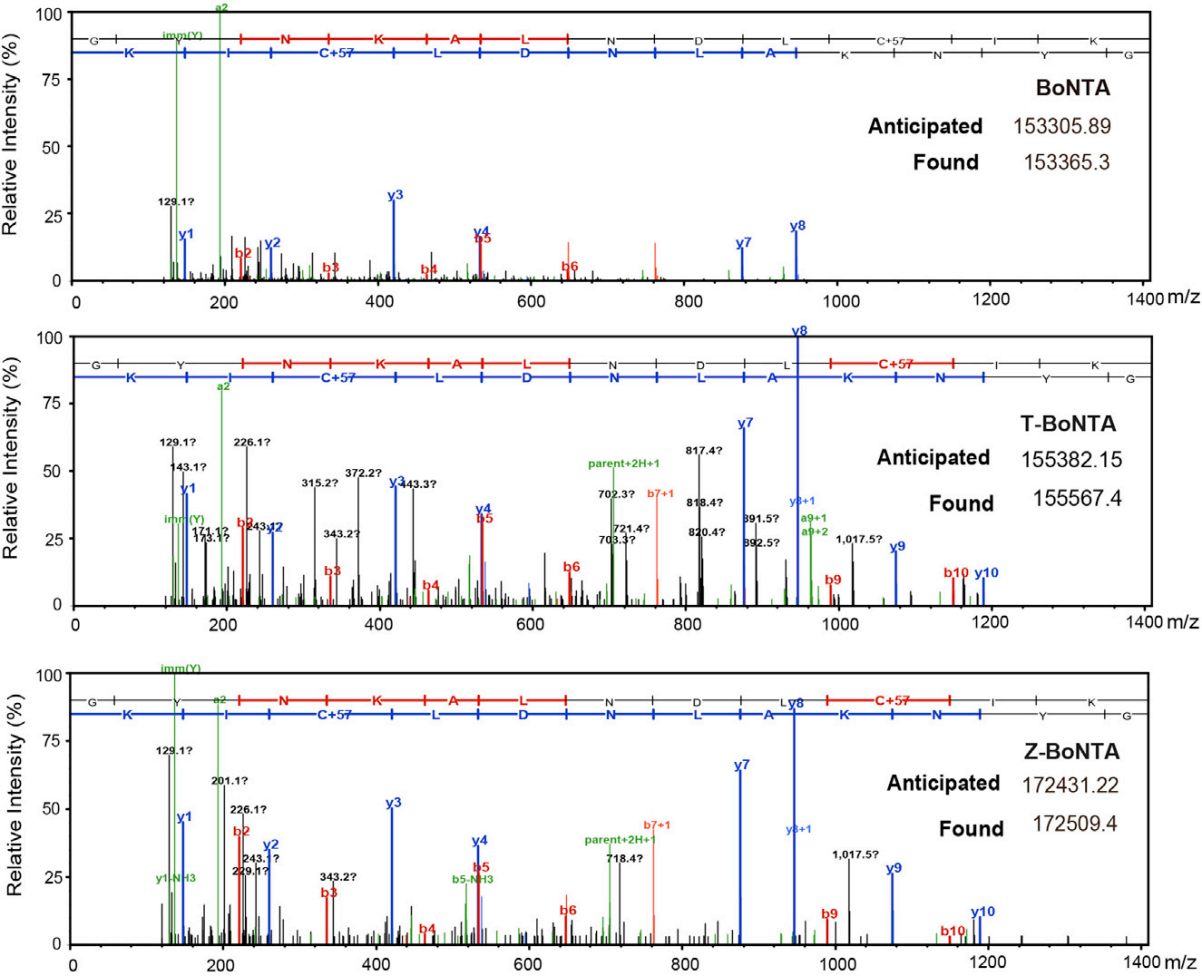
MS analysis of the molecular weight of CPP-BoNTA proteins

15. Mass spectrometry analyses of CPP-BoNTA proteins (Figure 4).
  a. Prepare 10–50 μg of protein samples with a purity of more than 90%.
  b. Use AB SCIEX TripleTOF™ 4600 coupled directly to a Nexera UHPLC LC-30A(SHIMADZU) with a 100 mm × 2.15 μm ACE C4 column for all LC-MS/MS analyses.
  c. Set the mobile phase A to be H_2_O with 0.1 FA, the mobile phase B to be CH_3_CN with 0.1 FA, and the flow rate to be 0.4 mL/min.
  d. Use Peakviewer software for database searching and spectral interpretation.

#### *In vitro* cleavage assay using SNAPtide

**Timing: 1 day**

***Note:*** In the SNAPtide, the N-terminal fluorophore is fluorescein isothiocyanate (FITC) and C-terminal quencher is 4-((4-(dimethylamino) phenyl) azo) benzoic acid (DABCYL). Once cleaved, the peptide will release the fluorophore FITC, generating fluorescence signal that can be measured spectroscopically.

**Figure 5:**
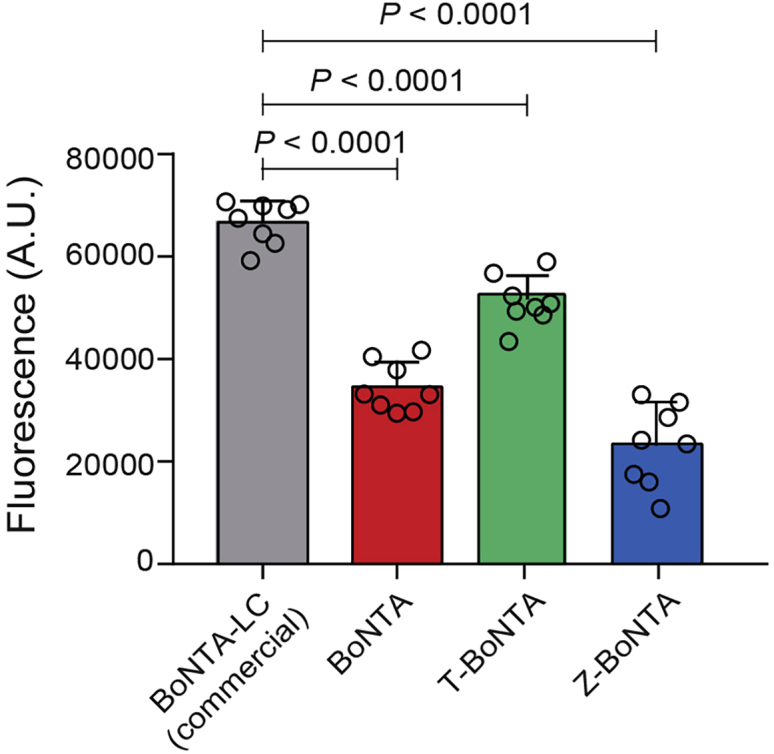
Evaluation of the *in vitro* activity of CPP-BoNTA proteins using SNAPtide assay Data are presented as mean ± standard deviation (SD). The significant difference between commercially available BoNTA-LC and home-purified BoNTA proteins (BoNTA, T-BoNTA or Z-BoNTA) is analyzed using Student’s t test. The p values are indicated.

16. Evaluation of the *in vitro* activity of CPP-BoNTA proteins (Figure 5).
  a. Prepare 96-well black microplate, and add reaction solution containing 1× SNAPtide assay buffer and 10 μM SNAPtide.
  b. Add CPP-BoNTA proteins to the reaction to a final concentration of 100 nM in a 100 μL reaction solution.
  c. Incubate the reaction at 37°C for 40 min.
  d. Measure the fluorescence using a plate reader with an excitation wavelength of 490 nm and an emission wavelength of 523 nm.

### *In vitro* analysis of the cell-penetrating activity of CPP-BoNTA in cell culture

**Timing: 3 days**

This section describes the procedures for analyzing the cell-penetrating activity of CPP-BoNTA in cell culture. Two major steps are involved including confocal imaging analysis and TissueFax cytometry-like analysis.

#### Analysis of treated N2a cells by confocal imaging

**Timing: 2 days (for step 17)**

**Figure 6:**
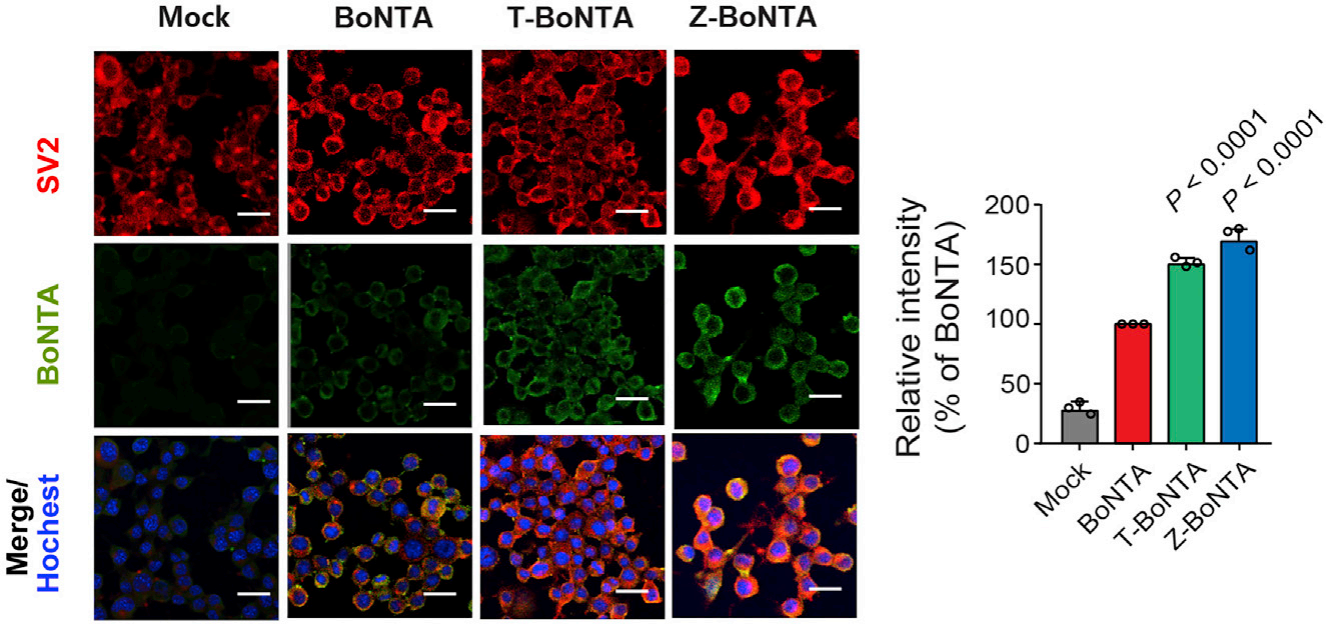
Evaluation of the cell-penetrating activity of CPP-BoNTA proteins in N2a cells using immunofluorescence staining Scale bar, 10 μm. The data in the bar plot are presented as mean ± SD. The significant differences between BoNTA and T-BoNTA or Z-BoNTA are analyzed using Student’s t test. The p values are indicated.

This step describes how to analyze the cell-penetrating activity of CPP-BoNTA by immunofluorescence staining and confocal imaging in mouse neuroblastoma N2a cells (Figure 6).

17. Mouse neuroblastoma N2a cells culture.
  a. Maintain N2a cells in MEM EARLES supplemented with 10% FBS, 1% MEM NEAA and 100 U/mL penicillin/streptomycin at 37°C in fully humidified atmosphere with 5% CO_2_.
  b. Coat 12 mm coverslips with 500 μL poly-L-lysine solution (1:10 dilution in water) by incubating at 25°C for 1 h in a 24-well plate.
  c. Wash coverslips three times with sterile PBS and air dry at 4°C for 12–16 h.
  d. Seed N2a cells on to poly-L-lysine-coated coverslips and culture in a 37°C/5% CO_2_ incubator for additional 2 days until a confluency of 70%–80% is reached.
  e. After 4 h incubation with 100 nM CPP-BoNTA, fix N2a cells with 4% paraformaldehyde (PFA) at 25°C for 15 min.
18. Permeabilization of N2a cells.
  a. Treat cells with PBS containing 0.1% Triton X-100 for 10 min.
  b. Wash cells three times with PBS containing 0.2% BSA.
19. Block non-specific binding site with PBS containing 0.2% BSA at 25°C for 30 min.
20. Primary antibody incubation.
  a. Stain cells (key resources table) with goat anti-FLAG (Novus) and rabbit anti-SV2A antibodies (Novus) at 1 to 200 dilution in PBS supplemented with 0.2% BSA.
  b. Cover plate at 25°C for 1–2 h during incubation.
  c. Wash the coverslips 5 times with PBS supplemented with 0.2% BSA.
  d. Wash the coverslips 5 times with PBS.
  e. Wash the coverslips 5 times with PBS supplemented with 0.2% BSA.
21. Secondary antibody incubation.
  a. Stain cells (key resources table) with PBS supplemented with 0.2% BSA and incubate with Alexa568-conjugated donkey anti-rabbit IgG (Life/Invitrogen) and Alexa488-conjugated donkey anti-goat IgG (Life/ Invitrogen) secondary antibodies.
  b. Cover plate at 25°C for 45 min during incubation.
  c. Wash the coverslips 5 times with PBS supplemented with 0.2% BSA.
  d. Wash the coverslips 5 times with PBS.
  e. Wash the coverslips 5 times with PBS supplemented with 0.2% BSA.

***Note:*** From this point on, the slices need to be kept in the dark. The slices can be stored at −80°C for three month.

22. Nuclei staining.
  a. Stain antibody-labeled cells sections with Hoechst 33342 (Invitrogen) for nucleus visualization.
  b. Cover plate at 25°C for 5–10 min during incubation.
  c. Wash the coverslips 3 times with PBS.
23. Mounting.
  a. Prepare microscope slides and add the coverslips on microscope slides with Prolong gold antifade reagent.
  b. Leave the microscope slides at 25°C to dry in the dark for 12–16 h.
  c. Store at 4°C for long-term use.
24. Confocal microscopy imaging.
  a. Visualize samples on a LSM710 laser scanning confocal microscopy (Carl Zeiss Microscopy GmbH, Jena, Germany) with the excitation/emission filters for red and green channels to be 493 nm/598 nm and 410 nm/507 nm respectively.
25. Measure the fluorescence intensity in each cell using ZEN 2011 imaging software (Zeiss).

***Note:*** Image analysis is carried out using the program FIJI. Background signal is subtracted from images using the Math function on the FIJI software.

#### TissueFAXS cytometry analysis

**Timing: 1 day (for step 26)**

This step describes the procedures for flow cytometry-like epifluorescence analysis using The TissueFAXS system. This approach is useful for quantification of a large set of samples.

**Figure 7:**
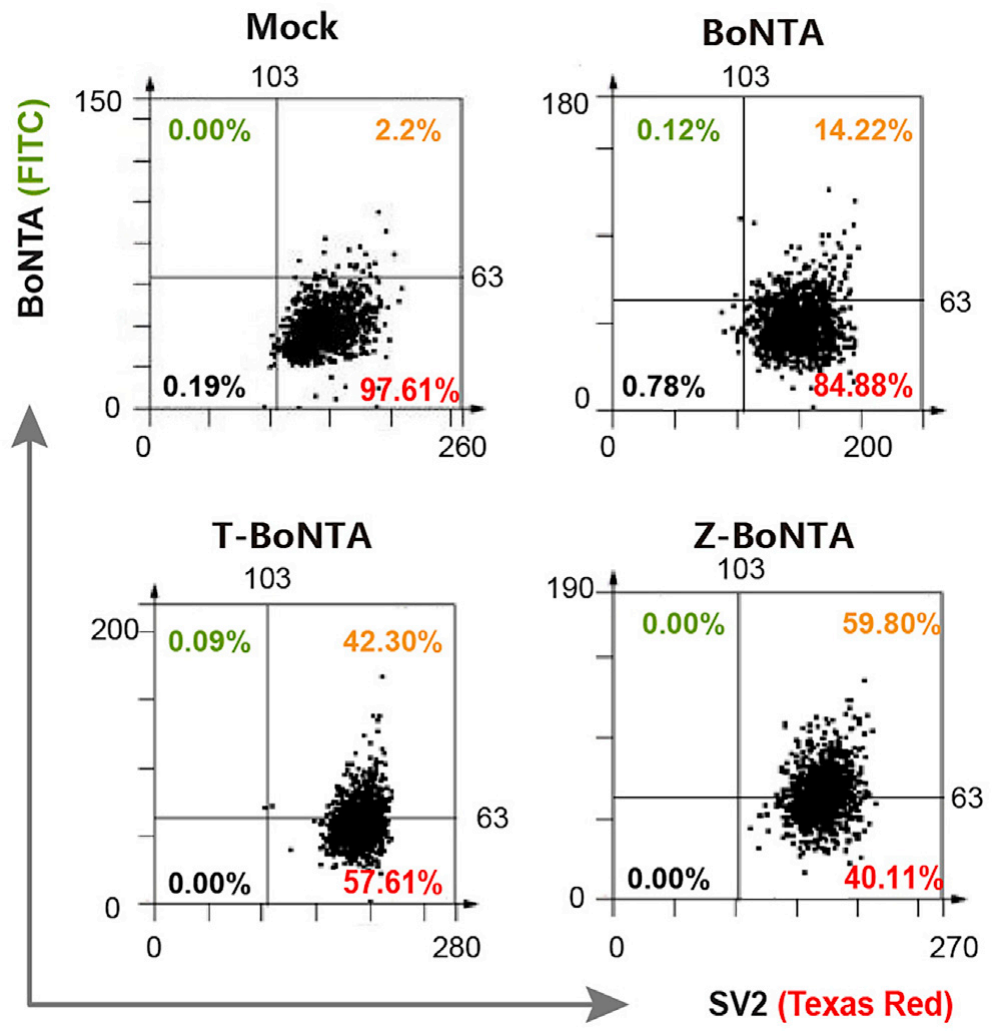
TissueFAXS cytometry analysis of co-expression of BoNTA proteins with SV2 receptors in mouse N2a cells

26. Visualize the prepared samples from step 23 on TissueFAXS (TissueGnostics, Vienna, Austria) fluorescence imaging system. Scan the whole section slices and calculate the fluorescence intensity using TissueQuest software (Figure 7).

### *In vivo* analysis of the efficacy and cell-penetrating activity of CPP-BoNTA in mice

**Timing: 7 days**

This section describes the procedures for the *in vivo* analyses of CPP-BoNTA in mice. Two major steps are included: analysis of the efficacy of CPP-BoNTA using digit abduction score, and analysis the cell-penetrating in gastrocnemius muscle.

#### Digit abduction score (DAS) assay

**Timing: 4 days (for step 27)**

In this step, we use digit abduction score (DAS) assay, which measures muscle-weakening effectiveness,^9^ to determine the pharmacologic activity (ED_50_) of BoNTA proteins. Therapeutic index is calculated using the determined ED_50_ and systemic toxicity (LD_50_).

**Figure 8:**
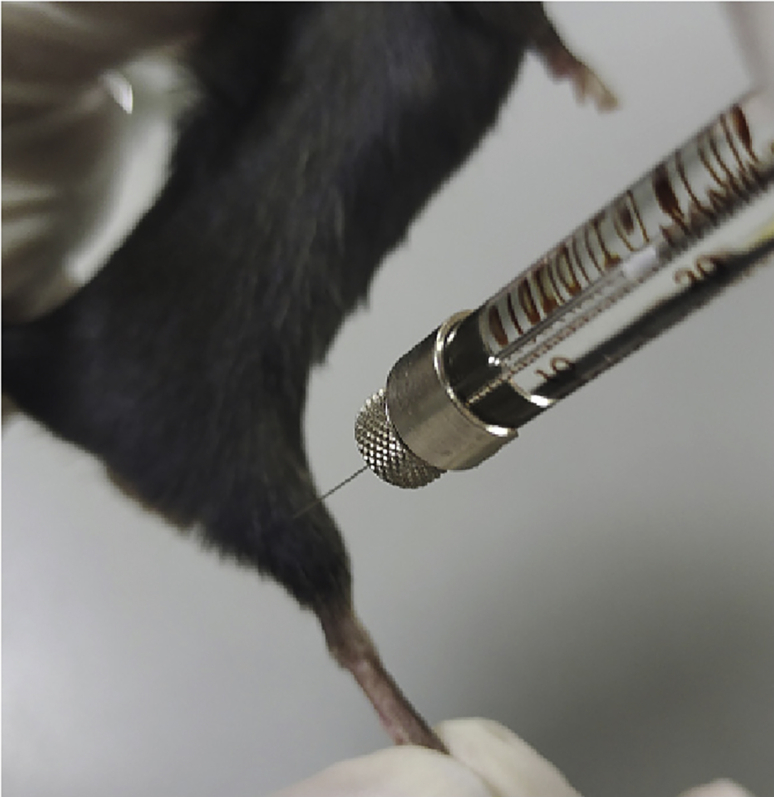
Injection of BoNTA proteins into the right gastrocnemius muscle of mice

***Note:*** It is preferred to include 5 or more mice for each dose to make the results more reliable.

27. Injection procedure (Figure 8).
  a. Prepare 8 to 12-week-old male or female C57BL/6 mice for intramuscular injection. For each experiment, inject at least five mice per dose (Table 1).

**Table 1:** The dosing and DAS results of BoNTA, T-BoNTA and Z-BoNTA in mice

BoNTA		T-BoNTA		Z-BoNTA	
Dose (ng/kg)	DAS	Dose (ng/kg)	DAS	Dose (ng/kg)	DAS
0.15	0.00 ± 0.0	0.05	0.20 ± 0.40	1.5	0.00 ± 0.00
0.5	0.20 ± 0.40	0.15	1.80 ± 0.98	5	2.20 ± 0.75
1.5	1.40 ± 0.49	0.5	2.60 ± 0.49	15	2.40 ± 0.80
5	2.40 ± 1.02	1.5	3.40 ± 0.49	50	3.00 ± 0.00
15	3.60 ± 0.49	5	4.00 ± 0.00	150	3.80 ± 0.40
50	4.00 ± 0.00	15	4.00 ± 0.00	300	4.00 ± 0.00
  b. Dispense different doses of CPP-BoNTA according to the body weight of the mice, so that the mice of each doses receive a fixed volume of 5 μL.
  c. Attach a 30-gauge needle to a sterile 250 μL Hamilton syringe.
  d. Inject CPP-BoNTA or vehicle (0.9% saline) into the head of the right gastrocnemius muscle of mice.

**CRITICAL:** Due to the small hind limbs of mice, it is necessary to avoid the needle to pass through the gastrocnemius muscle, which will result in ineffective injection.

**CRITICAL:** It is necessary to avoid bleeding during the injection, otherwise the mice may lick the wounds and swallow the injected proteins. If bleeding is caused, push the wound with an alcohol cotton ball to stop the bleeding and then wipe off the blood.

**Figure 9:**
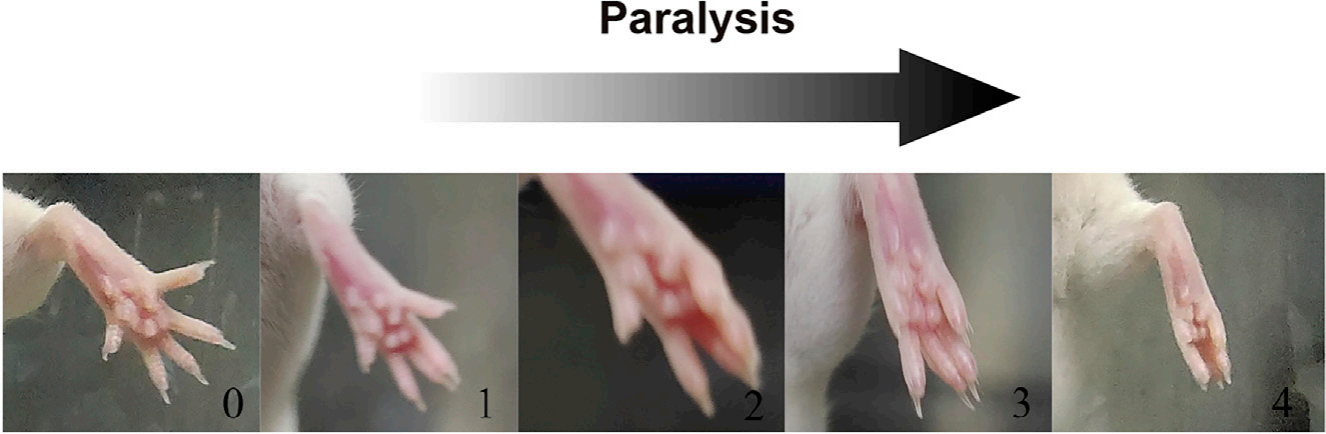
Representative DAS values The figure shows the various degree of digit abduction shown by mice.

28. Measurement of DAS score.
  a. At two days after injection, determine the DAS of each mouse.
  b. Suspend mice briefly by their tails to elicit a characteristic startle response in which the mice extend their hind-limbs and abduct their hind digits.
  c. Observe and record the shape of the foot and digits of the suspended mouse. The DAS values are assigned as follows^9^ (Figure 9):
    i. Zero-point: flat foot, digit spread same as the untreated leg.
    ii. One-point: flat foot, a difference in the width of digit abduction compared to the untreated leg or two digits touching and the rest spread completely.
    iii. Two-point: flat foot, slight space open at tips of all digits or three digits touching.
    iv. Three-point: five digits touching if foot is flat, or four digits touching if foot is curved.
    v. Four-point: curved foot, all five digits touching.

The doses and DAS results of BoNTA, T-BoNTA and Z-BoNTA proteins are summarized in Table 1.

**CRITICAL:** Two observers are required for DAS measurements to ensure that the data obtained are unbiased. The two observers must be blinded to the treatment, and at no time should they communicate with regard to the DAS values that they assign.

***Note:*** Data are shown as mean ± standard deviation (SD).

29. Determination of the systemic effects and therapeutic index.
  a. Determine the half intramuscular lethal dose (IMLD_50_).
    ***Note:*** IMLD_50_ is defined as the dose at which half of the mice die following treatment. Set the end point of monitoring at day 5, after which no further death can be found (Figure 10A).
    **Figure 10:**
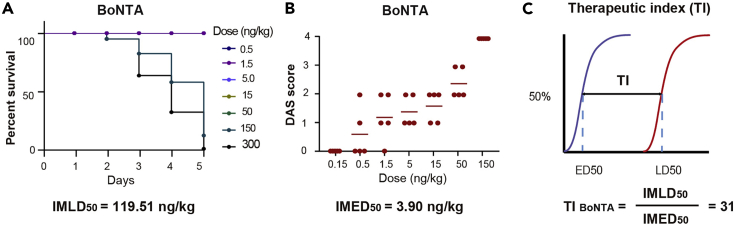
The therapeutic index of BoNTA proteins (A) Dose-dependent acute toxicity of BoNTA proteins. IM, intramuscular. (B) Dose-dependent muscle-paralyzing activity of BoNTA proteins. (C) The therapeutic index of BoNTA proteins.
  b. Determine the half intramuscular effective dose (IMED_50_) (Figures 9 and 10B).
  c. Calculate the intramuscular therapeutic index by dividing IMLD_50_ by IMED_50_ (Figure 10C).

#### Immunofluorescence analysis of treated gastrocnemius muscle

**Timing: 3 days (for step 30)**

In this step, the mice injected with CPP-BoNTA in the previous experiment will be sacrificed and the gastrocnemius muscle tissue is collected and immunofluorescence stained for evaluation of the cell-penetrating activity of CPP-BoNTA *in vivo*.

30. Sample Preparation.
  a. Section fresh gastrocnemius muscles into small pieces.
  b. Fix immediately with 4% paraformaldehyde at 25°C for 10 min.
  c. Dehydrate for 12–16 h in 30% sucrose.
  d. Dry the tissue blocks with paper towels.
  e. Place the tissue blocks on tissue molds.
  f. Incubate the tissue blocks with 100% optimal cutting temperature compound (OCT) over a total period of 4 h at −80°C.
  g. Frozen-section OCT-embedded gastrocnemius muscles serially at 10 μm interval along the transverse and longitudinal direction.
31. Blocking step.
  a. Block non-specific binding site with PBS supplemented with 5% BSA at 25°C for 1 h.
  b. Wash the sections 5 times with PBS for 5 min each time.
32. Primary antibody incubation.
  a. Incubate the slices with goat anti-FLAG antibody and anti-SV2A antibodies which are diluted 200-fold in PBS supplemented with 0.2% BSA at 4°C for 12–16 h.
  b. Wash the slices 3 times with PBS supplemented with 0.2% BSA.
  c. Wash the slices 3 times with PBS.
  d. Wash the slices 3 times with PBS supplemented with 0.2% BSA.
33. Secondary antibody incubation.
  a. Incubate with fluorochrome-conjugated secondary antibody which is diluted 1000-fold in PBS supplemented with 0.2% BSA for 1 h at 25°C in dark.
  b. Wash the slices 3 times with PBS supplemented with 0.2% BSA.
  c. Wash the slices 3 times with PBS.
  d. Wash the slices 3 times with PBS supplemented with 0.2% BSA.

***Note:*** From this point on, the slices need to be kept in the dark.

34. Nucleus Staining.
  a. Incubate with Hoechst 33342 which is diluted 5,000-fold in PBS for 10 min at 25°C in the dark.
  b. Wash the slices 3 times with PBS.
35. Mounting.
  a. Prepare microscope slides and add the coverslips on microscope slides with Prolong gold antifade reagent.
  b. Coat the edges of the coverslip with a thin layer of clear nail polish and leave to dry in the dark for 12–16 h.
  c. Store at 4°C for long-term use.

**Pause point:** The slides can be stored at 4°C for up to three months but prolonged storage may affect imaging resolution.

**Figure 11:**
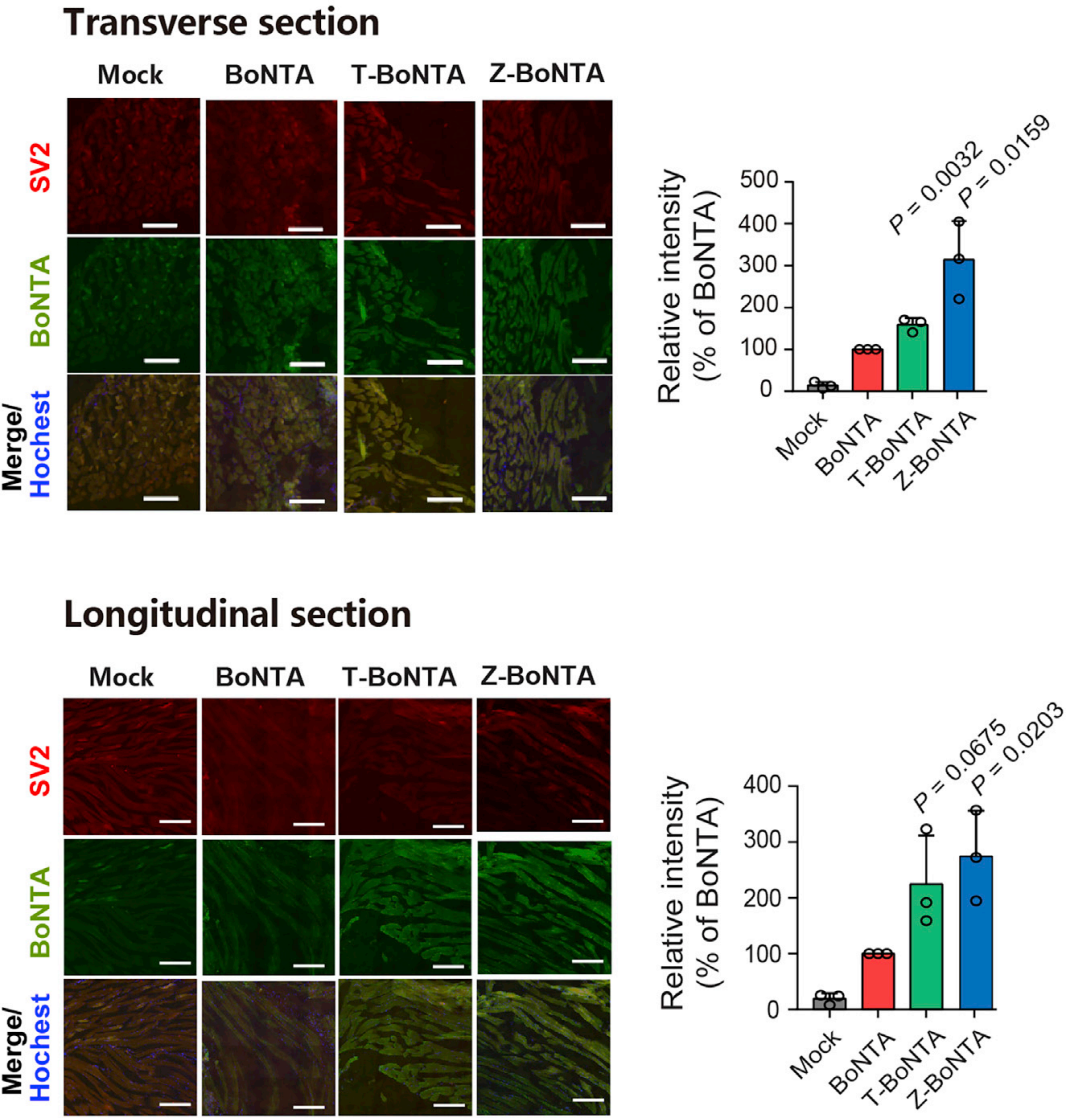
Evaluation of the *in vivo* cellular uptake of intramuscularly injected CPP-BoNTA proteins in mouse gastrocnemius muscles using immunofluorescence staining Scale bar, 200 μm. The data in the bar plot are presented as mean ± SD. The significant differences between BoNTA and T-BoNTA or Z-BoNTA are analyzed using Student’s t test. The p values are indicated.

36. Confocal microscopy imaging.
  a. Prepare microscope slides and add the coverslips on microscope slides with Prolong gold antifade reagent.
  b. Visualize the samples on a LSM710 laser scanning confocal microscopy (Carl Zeiss Microscopy GmbH, Jena, Germany), with the excitation/emission filters for red and green channels to be 493 nm/598 nm and 410 nm/507 nm respectively.
37. Measure the fluorescence intensity in each cell by ZEN 2011 imaging software (Zeiss) (Figure 11).

## Expected outcomes

BoNTA is widely used in treating neuromuscular disorders. Commercial BoNTA drugs are prepared by fermentation of *Clostridium botulinum*. Here we provide a step-by-step protocol for producing recombinant BoNTA proteins harboring cell-penetrating peptides using the BEVS-insect cell expression system. Following this protocol, highly homogenous proteins can be obtained (Figure 3). These proteins exhibited improved therapeutic index (Figure 10) and enhanced cellular uptake (Figure 11) in mice.

## Limitations

The *in vivo* activity of purified BoNTA proteins is determined using digit abduction score.^9^ While this method is the gold standard for academic and industrial applications, it is a semi-quantitative method that relies on the visual inspection from two researchers. This may generate variations under different experimental settings, such different researchers. Therefore, cautions should be taken when the results are interpreted. In addition, this protocol should be only used for academic scale protein production.

## Troubleshooting

### Problem 1

The concentration of isolated and purified bacmids are too low (step 8: Bacmid preparation).

### Potential solution

In the process of bacmid isolation, avoid the DNA fragmentation due to shearing force. In addition, pre-chilled isopropanol and 70% ethanol can increase the efficiency of DNA precipitation. Last, it is proper to leave some residual isopropanol or ethanol to avoid the disruption and loss of DNA sample given sufficient time is provided to air dry the residual isopropanol or ethanol. Generally, the concentration of purified bacmids should be more than 1,000 ng/μL.

### Problem 2

During bacmid transfection, Sf9 cells are contaminated with bacteria (step 9: Transfection of bacmid into Sf9 cells).

### Potential solution

Make sure sterile environment is maintained when removing 70% ethanol from the tube.

### Problem 3

During the purification step, a large amount of target protein remains in the cell pellet (step 12: Purification with Ni-NTA agarose beads).

### Potential solution

Increase the concentration of salt ions in the lysis buffer. We find that a solution containing 2 M NaCl can help improve the yield and purity of CPP-BoNTA proteins.

### Problem 4

When N2a cells are visualized by confocal microscopy, many cells are overlapped, making it difficult to observe fluorescence (step 17: Mouse neuroblastoma N2a cells culture).

### Potential solution

When the cell density is too high, N2a cells tend to aggregate rather than uniformly distributing in the coverslips. Therefore, attention should be paid during cell seeding process.

### Problem 5

During the immunofluorescence experiment, the fluorescence signals of the two channels interfere with each other, resulting in the confusion of the received fluorescence signals (steps 24 and 36: Confocal microscopy imaging).

### Potential solution

Fluorophore-conjugated secondary antibody used in different channels should be carefully selected to avoid overlap in the emission filters. When receiving the fluorescence signal with small emission filters, attention should be paid to adjust the range of the filters.

### Problem 6

During the DAS experiments, high frequency of injection failure or body injury are observed in mice due to improper experimental manipulations (step 27: Injection procedure).

### Potential solution

Hold the mouse by the tail and let it cling to the cage, so that its whole body is in a stretch state. And then press the mouse’s back with the palm of the hands while slowly moving your fingers from the back towards the neck with thumb and index finger. This can grasp the skin on the back of the mouse neck and thus calm down the mice for further experimentation.

## Resource availability

### Lead contact

Further information and requests for resources and reagents should be directed to and will be fulfilled by the lead contact, Jia Liu (liujia@shanghaitech.edu.cn (J.L.).

### Materials availability

Plasmids, primers, recombinant proteins, and any other research reagents generated by the authors will be distributed upon request to other research investigators under a material transfer agreement.

## Data Availability

This study did not generate or analyze datasets or code. The published article includes the figures generated with this protocol.
